# An approach to learn regulation to maximize growth and entropy production rates in metabolism

**DOI:** 10.3389/fsysb.2023.981866

**Published:** 2023-04-05

**Authors:** Ethan King, Jesse Holzer, Justin A. North, William R. Cannon

**Affiliations:** ^1^ Pacific Northwest National Laboratory, Richland, WA, United States; ^2^ Department of Microbiology, The Ohio State University, Columbus, OH, United States; ^3^ Interdisciplinary Center for Quantitative Modeling in Biology University of California, Riverside, CA, United States

**Keywords:** metabolism, regulation, maximum entropy, optimization, modeling

## Abstract

Elucidating cell regulation remains a challenging task due to the complexity of metabolism and the difficulty of experimental measurements. Here we present a method for prediction of cell regulation to maximize cell growth rate while maintaining the solvent capacity of the cell. Prediction is formulated as an optimization problem using a thermodynamic framework that can leverage experimental data. We develop a formulation and variable initialization procedure that allows for computing solutions of the optimization with an interior point method. The approach is applied to photoheterotrophic growth of *Rhodospirilium rubrum* using ethanol as a carbon source, which has applications to biosynthesis of ethylene production. Growth is captured as the rate of synthesis of amino acids into proteins, and synthesis of nucleotide triphoshaptes into RNA and DNA. The method predicts regulation that produces a high rate of protein and RNA synthesis while DNA synthesis is reduced close to zero in agreement with production of DNA being turned off for much of the cell cycle.

## 1 Introduction

Biological systems can be understood as dissipative systems analogous to tornadoes and hurricanes. Dissipative systems act by taking the most probable path to reduce the energy difference in the environment. These most probable paths result in cyclical patterns of material movement that act to transport the material from regions of high energy to regions of lower energy. In tornadoes, air movement becomes correlated and cyclical in which hot air is driven up and cool air down. Whether one considers entropy from a thermodynamic or information standpoint, the concept is the same—the most probable path allowed by physical constraints is taken. It is in this respect that we expect the dynamics of biological systems act to maximize their entropy production rates to the extent possible. In this regard, biological processes are highly constrained by physical and biological limitations—constraints that are critical for their function. Moreover, for biological systems, the adaptation of their dynamics to maximize their entropy production rates occurs on the time scale of natural selection. This is to say, through natural selection their genome structure and metabolism become updated such that they act to move to the most probable state possible by taking the least action path. On the shorter timescale of a cell’s lifetime, those adaptations also include complex mechansims for adapting to changing environmental conditions. These adaptation mechanisms serve to control the dynamics in such a way that their ability to carry out auto-catalysis (Hinshelwood, 1952) is preserved. Adaptation is accomplished through regulation of metabolic reaction pathways such that organisms remain viable in a physico-chemical sense despite rapid or dramatic shifts in the environment.

Because regulation is at the heart of understanding biological processes, discovery and understanding of the basis for regulation is critical to the field of biology. Regulation may be in the form of just-in-time regulation, graded regulation, switch-like regulation, pre-programmed regulation such as the circadian clock system, or even the choice to not regulate when it is too costly to alter the respective enzyme expression but to instead constitutively turn on an activity (Sivak and Thomson, 2014). Regulation is an important aspect of fitness in that organisms and even individual cells must regulate themselves so that they act as efficiently and as quickly as possible, otherwise they are outcompeted in their environment. Consequently, the environment over the time of evolution shapes regulation. Those cell phenotypes that operate in the most efficient manner in nutrient poor and dramatically shifting environments will tend towards fixation in the population. In rich environments in which selection pressure is not strong, dysregulation of cells can result in uncontrolled growth, manifest as cancer in metazoans.

Regulation in biological systems has been historically uncovered using experiments carefully designed to test hypotheses, typically employing isotope labeling methods to track reaction fluxes. Such meticulous but labor intensive studies have been quite successful at uncovering metabolic regulation (Larsson-Raznikiewicz, 1967; Newsholme and Start, 1973; Waygood and Sanwal, 1974; Waygood et al., 1975; 1976; Aithal et al., 1985; Jitrapakdee and Wallace, 1999; Holness and Sugden, 2003; Lehninger et al., 2005; Hallows et al., 2012; Saha et al., 2014; Wang et al., 2014), yet we are far from a complete understanding of regulation in even model organisms. High-throughput methods to uncover regulation are highly desirable, and recent work in this area has made progress (Hackett et al., 2016; Reznik et al., 2017), but we are still a long way from a routine method for determining regulation, either by experiment or prediction.

In a recent study by Hackett et al. (2016), Michealis-Menten steady-state kinetic models with regulation were used to predict reaction fluxes and concentrations. The predictions were compared to reaction fluxes inferred from 13C isotope experiments and concentrations derived from mass spectrometry and NMR measurements (Hackett et al., 2016). The correlation between simulation-predicted fluxes and experimentally-inferred fluxes were evaluated with and without regulation in the simulation. If the match was better with regulation, then regulation was assumed. The work was a *tour de force* in that 25 chemostat studies were used to carefully measure both absolute and relative metabolomics data while at the same time cover as much of the proteome as possible.

An approach used by Reznik et al. (2017), has less reliance on multimodal experimental designs, and instead used sophisticated informatics to develop a model of small molecule regulatory networks from curated databases of enzymes. They integrated the regulatory network with a metabolic model of *Escherichia coli*, and distilled information on how substrates and inhibitors contribute to metabolic flux regulation (Reznik et al., 2017).

More recently, Britton et al. (2020) have developed optimization and reinforcement learning methods that predict which enzymes need to be regulated to maintain metabolite concentrations at a level such that the diffusion process within the cell remains viable. These methods build on work by Cannon et al. (2018) that takes advantage of a maximum path-entropy/caliber (Jaynes, 1985; Dewar, 2009; Dixit et al., 2018) formulation of the law of mass action in order to predict likely metabolite concentrations. The advantage of these approaches is that minimal measurements and few experimentally-derived parameters are required to predict regulation.

Maximum entropy methods are attractive because they use the most likely values of the parameters needed to model a process (Jaynes, 1985; Dewar, 2009; Dixit et al., 2018). That is, rather than a large search through parameter space to determine the most likely kinetic parameters, a maximum entropy formulation can provide the solution directly, then if needed, reaction parameters can be back calculated. Strictly speaking, these methods maximize the path entropy of a system, meaning that they maximize the entropy subject to the stoichiometric constraints imposed by the reactions and the constraints due to the system boundary conditions. Without the stoichiometric constraints, each molecular species would move to its natural abundance determined by its standard chemical potential. Without non-equilibrium boundary constraints, the reactions would all go to the equilibrium state.

In the study by Britton et al. (2020), regulation was inferred only with regard to which reactions needed to be controlled to keep metabolites at concentrations that are not so high that they would impede diffusion (Britton et al., 2020). As mentioned above, in an environmental niche with finite nutrient resources for growth, for cells to persist they must replicate in a timely and efficient manner in order to compete with other species. Therefore in the context of evolution we expect to see regulation in cells that maximizes growth, and only produces those enzyme catalysts that contribute to growth in a timely and efficient manner.

In this study, we develop an optimization approach that allows for extension of the work by Britton et al. (2020) to be applied for more general constraint based modeling, and demonstrate our method for prediction of regulation that maximizes growth pathways while still constraining metabolite levels to physiological values. While flux balance type analyses optimize a similar objective and may include information about regulation (Covert et al., 2001) or even thermodynamic feasibility constraints (Henry et al., 2007), the method we present is fundamentally different in that we use a thermodynamic perspective to directly infer the most likely distribution of both fluxes and metabolite concentrations subject to given constraints.

We apply our method, that we term pathway-controlled optimization (PCO), to the metabolism and enzymatic activities of *Rhodospirilium rubrum*, a purple non-sulfur photosynthetic bacterium that is being used as a synthetic biology organism for the purpose of ethylene production (North et al., 2020). We compare the regulation and reaction fluxes predicted by this new method to the approach presented in (Britton et al., 2020) that adjust regulation only to maintain metabolite concentrations at physiological levels.

## 2 Methods

We begin this section with a brief introduction to the maximum path entropy approach developed in (Cannon et al., 2018) and (Britton et al., 2020). Then we present how we extend this approach to account for regulation in cells to optimize growth.

### 2.1 Maximum path entropy solution without regulation

For a set of molecular species I={A,B,C,D} having respective concentrations *n*_*i*_ for each species i∈I , participating in a set of reactions J={1,−1} , with unsigned stoichiometric coefficients *ν*_*i*,*j*_ for each molecular species *i* and each reaction j∈J , the reversible reaction is described by the chemical equation,

$$\nu_{A,1}n_{A}+\nu_{B,1}n_{B}\overset{k_{1}}{\underset{k_{-1}}{⇌}}\nu_{C,1}n_{C}+\nu_{D,1}n_{D}.$$ (1)

Here, *k*_1_ and *k*_−1_ are the reaction rate parameters.

Let |I| denote the size of the set I and let $n\in R^{\left|I\right|}$ be the vector of molecular counts with elements *n*_*i*_. If *S* is the stoichiometric matrix of elements *S*_*i*,*j*_ = *γ*_*i*,*j*_ where *γ*_*i*,*j*_ are the signed stoichiometric coefficients such that *ν*_*i*,*j*_ = |*γ*_*i*,*j*_|, we have according to the law of mass action,

$$\frac{dn}{dt}=SJ\left(n\right)$$ (2)

where for a given *n*, *J*(*n*) is the net flux of the set of forward and reverse reactions. The chemical species occurring on the left hand side of the equation are known as reactants and belong to the subset $I_{R_{j}}\subset I$ , while those on the right hand side are known as products and belong to the subset $I_{P_{j}}\subset I$ .

For any reversible reaction with forward and reverse reactions + *j* and −*j*, the net flux is given by,

$$J_{j}\left(n\right)\;=\;k_{j}\underset{i\in I_{R_{j}}}{\prod }n_{i}^{\nu_{i,j}}-k_{-j}\underset{i\in I_{R_{-j}}}{\prod }n_{i}^{\nu_{i,-j}}.$$ (3)

Eq. 3 is a purely kinetic description of the reaction flux. For elementary reactions, thermodynamic terms are introduced into the law of mass action by a simple factorization

$$J_{j}\left(n\right)=\;k_{-j}\underset{i\in I_{R_{-j}}}{\prod }n_{i}^{\nu_{i,-j}}\left(\frac{k_{j}\prod_{i\in I_{R_{j}}}n_{i}^{\nu_{i,j}}}{k_{-j}\prod_{i\in I_{R_{-j}}}n_{i}^{\nu_{i,-j}}}\right)-k_{j}\underset{i\in I_{R_{j}}}{\prod }n_{i}^{\nu_{i,j}}\left(\frac{k_{-j}\prod_{i\in I_{R_{-j}}}n_{i}^{\nu_{i,-j}}}{k_{j}\prod_{i\in I_{R_{j}}}n_{i}^{\nu_{i,j}}}\right)$$ (4)

$$=\;k_{-j}\underset{i\in I_{R_{-j}}}{\prod }n_{i}^{\nu_{i,-j}}\left(K_{j}\underset{i}{\prod }n_{i}^{\gamma_{i,j}}\right)-k_{j}\underset{i\in I_{R_{j}}}{\prod }n_{i}^{\nu_{i,j}}\left(K_{-j}\underset{i}{\prod }n_{i}^{\gamma_{i,-j}}\right),$$ (5)

where $K_{j}=\frac{k_{j}}{k_{-j}}$ is the equilibrium constant for reaction *j*. In Eq. 5, the terms in parentheses are related to the thermodynamic forces on the reaction while the terms outside the parentheses are the time-dependent kinetic terms. Setting the latter terms to a constant gives the Marcelin Equation (Marcelin, 1910), in which each forward and reverse reaction occurs on the same timescale by postulate. While this postulate is incorrect for determining the true dynamics of the system, the Marcelin formulation of Eq. 5 does give a way to the maximum path entropy solution (Cannon et al., 2018; Britton et al., 2020) by assuming that all reactions occur on the same timescale and that their fluxes are proportional to the thermodynamic forces on them. Once these fluxes and concentrations are known from the maximum path entropy solution, they can be used to back-calculate the rate constants that will give the corresponding true dynamics (Cannon et al., 2018; Britton et al., 2020).

Using the maximum path entropy solution, and representing the thermodynamic reaction forces for each reaction j∈J as,

$$f_{j}\left(n\right)=K_{j}\underset{i\in I}{\prod }n_{i}^{-\gamma_{i,j}},$$ (6)

the flux is then given by

$$J_{j}\left(n\right)=f_{j}\left(n\right)-\frac{1}{f_{j}\left(n\right)}$$ (7)

and we can express the time dependence of each metabolite i∈I as follows

$$\frac{dn_{i}}{dt}=\underset{j\in J}{\sum }\gamma_{i,j}\left(f_{j}\left(n\right)-\frac{1}{f_{j}\left(n\right)}\right).$$ (8)

### 2.2 Steady state

At the maximum path entropy configuration the system is in a steady state, where a set of metabolites, denoted $I_{f}$ , are assumed to be held at fixed concentrations as boundary conditions for the system. For the rest of the metabolites, $I_{v}=I\backslash I_{f}$ , their steady state concentration is free and their rate of change is zero. For all $i\in I_{f}$ , let ${\bar{n}}_{i}$ be the fixed concentration of the metabolite. A steady state is defined as a solution to the following system of equations,

$$\begin{array}{cc} & \frac{dn_{i}}{dt}\;=\;0\;\;\forall i\in I_{v},\\ & n_{i}\;=\;{\bar{n}}_{i}\;\;\forall i\in I_{f}. \end{array}$$ (9)

### 2.3 Controlling metabolite concentrations

Metabolite concentrations predicted using the maximum path entropy approach without regulation will produce values that are much too large to be physiologically reasonable—concentrations may approach the limit of their solubility, causing the cytoplasm to become glass-like (Parry et al., 2014; Heimlicher et al., 2019).

That regulation is needed to control concentrations *in vivo* was proposed early in the field of enzymology (Atkinson, 1969;Atkinson, 1977). In a previously reported method to control concentrations (Britton et al., 2020), Britton, *et al.* applied regulation to reactions using a scalar valued activity coefficient *α*_*j*_ ∈ [0.0, 1.0] for each reaction *j*, that linearly scales the reaction rate such that the time dependence of metabolite concentration *n*_*i*_ is given by,

$$\frac{dn_{i}}{dt}=\underset{j\in J}{\sum }\gamma_{i,j}J_{j}\left(n,\alpha_{j}\right),$$ (10)

where

$$J_{j}\left(n,\alpha_{j}\right)=\alpha_{j}\left(f_{j}\left(n\right)-\frac{1}{f_{j}\left(n\right)}\right).$$ (11)

When *α*_*j*_ = 1.0, the reaction is fully active and when *α*_*j*_ = 0.0, the activity, and hence reaction rate *J*_*j*_ (*n*, *α*_*j*_), is zero. Activities are adjusted in a deterministic manner by characterizing the sensitivity of a metabolite concentration to an enzyme activity using Metabolic Control Analysis (MCA) (Sauro, 2018). In MCA, the concentration control coefficient *C*_*i*,*j*_ measures the sensitivity of a concentration *n*_*j*_ to an activity coefficient *α*_*j*_,

$$C_{i,j}=\frac{\partial \log n_{i}}{\partial \log\alpha_{j}}.$$ (12)

Using this approach, metabolites whose predicted concentrations are furthest from their experimentally observed values, as measured by the log ratio of the predicted to observed concentration, are reduced first. The concentration of a metabolite *n*_*i*_ is reduced by adjusting the activity coefficient *α*_*j*_ that has the largest influence on *n*_*i*_ and all other metabolites that exceed their experimentally observed concentrations, as determined by the sensitivity analysis. This process is iteratively carried out until all concentrations are at or below the experimentally observed concentrations. Details of the approach are provided in the study by Britton et al. (2020). In the cases discussed herein, the maximum value of the experimentally observed metabolite concentrations are taken to be *n*_*i*_ = 1.0 mM for $i\in I_{v}$ and for $i\in I_{f}$ the values are taken from mass spectrometry observed concentrations for bacteria (Bennett et al., 2009; Park et al., 2016).

### 2.4 Controlling metabolite concentrations and maximizing growth

While controlling metabolite concentrations may be a primary role of metabolic regulation, natural selection also requires that organisms be regulated to grow fast and efficiently—using the available energy from the environment to ensure survival and compete with others. Here we develop a novel approach, we call pathway-controlled optimization (PCO), whose goal is to obtain this biological objective, and derive a formulation that allows for tractable numerical solutions.

Let G⊂J be the set of reactions corresponding to production of biomass. We formulate the steady state with maximum biomass production as the solution of the optimization problem.

$$\max\underset{j\in G}{\sum }J_{j}\left(n,\alpha_{j}\right)$$ (13a)

$$\begin{array}{ll} & \text{subject to:}\\ & \frac{dn_{i}}{dt}\;=\;0\;\;\forall \;i\in I_{v}, \end{array}$$ (13b)

$$n_{i}\;=\;{\bar{n}}_{i}\;\;\forall \;i\in I_{f},$$ (13c)

$$0\leq n_{i}\leq n_{\max}\;\;\forall \;i\in I_{v},$$ (13d)

$$0\leq \alpha_{j}\leq 1\;\;\forall \;j\in J.$$ (13e)

The objective seeks to maximize the flux through the growth reactions G while the constraints (13b) - (13c) ensure that the steady state (9) is satisfied. The activity coefficients and metabolite concentrations are further restricted to physiologically meaningful values with the constraints (13e) and (13d).

The formulation of the PCO problem is simple to express but difficult to solve. The steady state constraints (13b) are non-linear and non-convex presenting significant challenges to optimization. Values for the flux, activity coefficients, and metabolite concentrations can also vary over many orders of magnitude, which introduces additional difficulty in employing numerical methods to compute solutions. In this work we present a more computationally tractable reformulation of the constraints and present numerical solutions from an interior point solver.

#### 2.4.1 Representing the steady state condition

To simplify solving the optimization, we reformulate the steady state constraint (13b) to be more numerically tractable. Let $S_{v}\in R^{\left|I_{v}|,|J\right|}$ be the sub-matrix of *S* with the rows corresponding to only the variable metabolites. Then at a steady state the reaction fluxes must satisfy

$$S_{v}J\left(n,\alpha \right)=0,$$ (14)

that is $J\left(n,\alpha \right)\in N\left(S_{v}\right)$ , where $N\left(S_{v}\right)$ is the nullspace of *S*_*v*_ defined

$$N\left(S_{v}\right)=\left\{x\in R^{\left|J\right|}\;:\;S_{v}x=0\right\}.$$ (15)

We separate (14) into the identification of a vector of fluxes $y\in R^{\left|J\right|}$ which satisfy steady state and the construction of a vector of metabolites $n\in R^{\left|I\right|}$ which achieve those fluxes with the two conditions,

$$\begin{array}{cc} y & \in N\left(S_{v}\right),\\ y_{j} & =J_{j}\left(n,\alpha_{j}\right)\;\;\forall \;j\in J. \end{array}$$ (16)

The expression *y*_*j*_ = *J*_*j*_ (*n*, *α*_*j*_) can be made more tractable by considering the computation of the reaction flux in terms of the log of the metabolites. For $\eta \in R^{\left|I\right|}$ let

$$\eta_{i}=\log\left(n_{i}\right)\;\;\forall \;i\in I,$$ (17)

then we have from (7) that the flux as a function of *η* in the maximum path entropy formulation is given by

$$J_{j}\left(\eta ,\alpha_{j}\right)=\alpha_{j}K_{j}e^{-\langle \;{\left(S\right)}_{j},\eta \;\rangle }-\alpha_{j}\frac{1}{K_{j}}e^{\langle \;{\left(S\right)}_{j},\eta \;\rangle },$$ (18)

where (*S*)_*j*_ is the *j*th column of *S*, and ⟨⋅, ⋅⟩ is the standard inner product. It follows that in terms of the log of the metabolite counts, conditions (16) are equivalent to.

$$y\in N\left(S_{v}\right),$$ (19a)

$$S^{T}\eta =\hat{y}\left(y,\alpha \right),$$ (19b)

$${\hat{y}}_{j}\left(y,\alpha \right)=\log\left(\frac{K_{j}}{2\alpha_{j}}\left(-y_{j}+\sqrt{y_{j}^{2}+4\alpha_{j}^{2}}\right)\right)\;\;\forall \;j\in J.$$ (19c)

Computation of $\hat{y}\left(y,\alpha \right)$ is prone to error. For example, in the instance where *y*_*j*_ is positive, rounding may result in evaluating the log of zero. To avoid these issues we use the following equivalent expression to (19c)

$${\hat{y}}_{j}\left(y,\alpha \right)=\log\left(K_{j}\right)+\mathrm{sgn}\left(y_{j}\right)\left(\log\left(2\alpha_{j}\right)-\log\left(|y_{j}|+\sqrt{y_{j}^{2}+4\alpha_{j}^{2}}\right)\right),$$ (20)

with sgn the signum function given by

$$\mathrm{sgn}\left(x\right)=\left\{\begin{array}{cc} 1\; & \;x>0\\ 0\; & \;x=0\\ -1\; & \;x<0 \end{array}\right..$$ (21)

A further complication in using the steady state conditions (19) as constraints for optimization is that the value of $\hat{y}\left(y,\alpha \right)$ can become extremely sensitive to small changes in the flux. In particular the partial derivative

$$\frac{\partial {\hat{y}}_{j}}{\partial y_{j}}=\frac{1}{\sqrt{y_{j}^{2}+4\alpha_{j}^{2}}}$$ (22)

grows unbounded as *α*_*j*_ and *y*_*j*_ go to zero. In this application we consistently found solutions with *α*_*j*_ and *y*_*j*_ on the order of 10^–9^ for some reactions while others were unregulated with values of *y*_*j*_ on the order of 10^3^. These discrepancies in scale are challenging for numerical methods.

However, it is not necessary to explicitly compute the activity coefficients in order to characterize a steady state. Instead it is sufficient only to identify metabolite concentrations that produce flux values that can be reduced to a steady state. We can capture this criteria with the conditions

$$\begin{array}{cc} & |y_{j}|\leq |J_{j}\left(n,1\right)|\;\;\forall \;j\in J,\\ & y_{j}J_{j}\left(n,1\right)>0, \end{array}$$ (23)

where **1** is the vector of all ones corresponding to no regulation for all reactions. If (23) is satisfied then any reaction fluxes *J*_*j*_ (*n*, **1**) with magnitude greater than the steady state value *y*_*j*_ can always be reduced to satisfy equality so long as they have the same sign, which is captured by the second condition in (23). This idea can also be captured in our modified formulation (19) for the steady state conditions.

For metabolites *η* and steady state fluxes *y* there exists an *α* such that (19) holds if the following hold

$$\begin{array}{cc} & |g_{j}-\log\left(K_{j}\right)|\geq -\left(\log\left(2\right)-\log\left(|y_{j}|+\sqrt{y_{j}^{2}+4}\right)\right)\;\;\forall \;j\in J,\\ & \left(\log\left(K_{j}\right)-g_{j}\right)y_{j}\geq 0\;\;\forall \;j\in J, \end{array}$$ (24)

where *g* = *S* ^*T*^ *η*. This follows from (19b)-(19c), (20), and the inequality

$$\log\left(2\alpha_{j}\right)-\log\left(|y_{j}|+\sqrt{y_{j}^{2}+4\alpha_{j}^{2}}\right)\leq \log\left(2\right)-\log\left(|y_{j}|+\sqrt{y_{j}^{2}+4}\right),$$ (25)

for all $y_{j}\in R$ and *α*_*j*_ ∈ [0, 1].

Therefore we can formulate steady state conditions equivalent to (19) without the need to explicitly specify the activity coefficients as follows.

$$y\in N\left(S_{v}\right),$$ (26a)

$$g=S^{T}\eta ,$$ (26b)

$$h_{j}=\mathrm{sgn}\left(y_{j}\right)\left(\log\left(2\right)-\log\left(|y_{j}|+\sqrt{y_{j}^{2}+4}\right)\right)\;\;\forall \;j\in J,$$ (26c)

$$|g_{j}-\log\left(K_{j}\right)|\geq |h_{j}|\;\;\forall \;j\in J,$$ (26d)

$$\left(\log\left(K_{j}\right)-g_{j}\right)y_{j}\geq 0\;\;\forall \;j\in J.$$ (26e)

#### 2.4.2 Numerical formulation of the steady state constraints

Implementing conditions (26a) - (26e) as constraints for numerical optimization requires further reformulation. The constraint (26d) is non-convex, and represents an ‘or’ condition depending on the sign of the flux for each reaction. This switching condition makes the feasible set non-convex and therefore challenging for optimization methods to search over. To find solutions we use a big *M* relaxation of (26d) with the constraints

$$h_{j}=\mathrm{sgn}\left(y_{j}\right)\left(\log\left(2\right)-\log\left(|y_{j}|+\sqrt{y_{j}^{2}+4}\right)\right)\;\;\forall \;j\in J,$$ (27a)

$$g_{j}-\log\left(K_{j}\right)\geq h_{j}-u_{j}M\;\;\forall \;j\in J,$$ (27b)

$$g_{j}-\log\left(K_{j}\right)\leq h_{j}+\left(1-u_{j}\right)M\;\;\forall \;j\in J,$$ (27c)

$$2u_{j}-1=\mathrm{sgn}\left(y_{j}\right)\;\;\forall \;j\in J,$$ (27d)

where *M* is taken to be a large constant. For each reaction the variable *u*_*j*_ is a “switching” term that relaxes either the constraint (27b) or (27c) depending on the sign of *y*_*j*_ such that only the correct constraint is imposed for each reaction.

In several constraints we utilize the signum function of the flux which is a discrete function, whereas the optimization method we use here requires continuous functions of all variables for the constraints. Therefore we approximate the signum function with

$$s\tilde{g}n\left(x\right)=\frac{\lambda x}{\lambda |x|+\epsilon }$$ (28)

for scaling parameters *λ* and *ϵ*.

To implement the constraint $y\in N\left(S_{v}\right)$ we construct a basis for the nullspace. For *m* the dimension of $N\left(S_{v}\right)$ , let $B\in R^{|J|,m}$ be a matrix with columns given by a basis of $N\left(S_{v}\right)$ . Then we have that

$$N\left(S_{v}\right)=\left\{B\beta \;:\;\beta \in R^{m}\right\},$$ (29)

and we capture the constraint $y\in N\left(S_{v}\right)$ with the expression *y* = *Bβ* for a $\beta \in R^{m}$ .

#### 2.4.3 Maximum growth optimization numerical formulation

Using the steady state conditions as outlined in sections 2.4.2 and 2.4.1 we formulate the maximum growth pathway-controlled optimization problem as

$$\underset{y,\eta }{\max}\;\underset{j\in G}{\sum }y_{j}$$ (30a)

$$\begin{array}{ll} \text{subject to:} & \\ y=B\beta , \end{array}$$ (30b)

$$g=S^{T}\eta ,$$ (30c)

$$h_{j}=s\tilde{g}n\left(y_{j}\right)\left(\log\left(2\right)-\log\left(|y_{j}|+\sqrt{y_{j}^{2}+4}\right)\right)\;\;\forall \;j\in J,$$ (30d)

$$g_{j}-\log\left(K_{j}\right)\geq h_{j}-uM\;\;\forall \;j\in J,$$ (30e)

$$g_{j}-\log\left(K_{j}\right)\leq h_{j}+\left(1-u\right)M\;\;\forall \;j\in J,$$ (30f)

$$\left(\log\left(K_{j}\right)-g_{j}\right)y_{j}\geq 0\;\;\forall \;j\in J,$$ (30g)

$$2u_{j}-1=s\tilde{g}n\left(y_{j}\right)\;\;\forall \;j\in J,$$ (30h)

$$\eta_{i}\leq \eta_{\max_{i}}\;\;\forall \;i\in I_{v},$$ (30i)

$$\eta_{i}={\bar{\eta }}_{i}\;\;\forall \;i\in I_{f},$$ (30j)

where the constraints (30b)-(30h) represent the steady state constraint (13b). Note that the activity coefficients *α* for reactions are not explicitly included, however for a solution with optimal log metabolite counts η^*^ and fluxes y^*^, the associated activity coefficients can be recovered as

$$\alpha_{j}^{*}=\frac{y_{j}^{*}}{J_{j}\left(\eta^{*}\right)}\;\;\forall \;j\in J.$$ (31)

It is important here to note that when (30) admits solutions they are not necessarily unique. For instance, those reactions that do not directly impact the objective may have a range of choices for the fluxes and constituent metabolite values that satisfy constraints (30b)–(30h) without changing the objective value. In particular constraints (30e) and (30f) are not neccesairly all that restrictive.

#### 2.4.4 Initialization for optimization

The greatest difficulty in solving (30) is in identifying the correct sign for the fluxes. If the correct flux directions are known then the problem simplifies considerably. Using IPOPT, a general non-linear interior point solver (Wächter and Biegler, 2006), we found that the solution and convergence of the solver was extremely sensitive to the variable initialization, as is common for non-convex problems. In particular, starting with fluxes close to the optimal directions was crucial for convergence to a good solution. To initialize the variables we solve for metabolite values that produces fluxes close to the gradient direction of the objective. Using random or other initialization methods we found that the solver failed to converge, or converged to points with smaller objective values.

To compute initial variable values we first find the projection of the gradient of the objective onto the space of steady state solutions, with

$$y_{g}=P_{N\left(S_{v}\right)}\left(\nabla_{y}\left[\underset{j\in G}{\sum }y_{j}\right]\right)$$ (32)

where $P_{N\left(S_{v}\right)}$ is the projection operator onto $N\left(S_{v}\right)$ . We then solve the constrained linear least squares problem

$$\underset{\eta }{\min}\;{\left|\left|\mathrm{sgn}\left(-y_{g}\right)◦\left(\;S^{T}\eta -\log\left(K\right)\;\right)-s\right|\right|}^{2}$$ (33a)

$$\begin{array}{cc} \text{subject to:} & \\ s_{j}\geq 0,\;\forall \;j\in J, \end{array}$$ (33b)

$$\eta_{i}\leq \eta_{\max_{i}}\;\;\forall \;i\in I_{v},$$ (33c)

$$\eta_{i}={\bar{\eta }}_{i}\;\;\forall \;i\in I_{f},$$ (33d)

where *s* is a vector of positive slack variables and ◦ is the Hadamard product. Consider that if the objective is zero at a solution *η*∗ then sgn (−*y*_*g*_) = sgn (*S* ^*T*^ *η*∗ − log(*K*)) which implies that there exists a *γ* > 0 such that (*η*∗, *γy*_*g*_) is a feasible point of (30). Therefore this is a feasible point with fluxes in the directions of *y*_*g*_. In practice solutions of (33) may only be close to zero, so we choose initial fluxes *y*∗ as

$$y^{*}=-\zeta P_{N\left(S_{v}\right)}\left(\;S^{T}\eta^{*}-\log\left(K\right)\;\right)$$ (34)

for a *ζ* > 0. Larger zeta values move the fluxes further from the origin and was found to improve convergence, likely as it reduces the number of fluxes that will switch sign early in the optimization.

### 2.5 Numerical solution

We solved (33) with the SciPy version 1.7.1 lsq_linear routine using the default configuration (Virtanen et al., 2020). A solution of (30) was then computed with IPOPT version 3.14.4 using Pyomo (Hart et al., 2017) again using the default optimization parameters except with tol = 1.0 × 10^−7^ and maximum iterations set to 10,000.

The parameters for $\tilde{\mathrm{sgn}}$ in constraint (30d) were *λ* = 1.0 × 10^50^ and *ϵ* = 1.0 × 10^−50^, while in constraint (30h) they were *λ* = 1 and *ϵ* = 1 × 10^−50^. The big *M* value was set to 100, and we used *ζ* = 10 for the initialization computation.

For all variable metabolites the upper bound was $\eta_{\max_{i}}=13.308$ . Values for the fixed metabolite concentrations, equilibrium constants, and stochiometeric matrix are given in the Supplementary Material. Equilibrium constants were computed using eQuilibrator version 0.3.1 (Beber et al., 2021). Values for the fixed metabolites were taken from mass spectrometry observed concentrations for bacteria (Bennett et al., 2009; Park et al., 2016).

### 2.6 Metabolic model

The metabolic model of *R. rubrum* consisted of 184 reactions and 204 metabolites including central metabolism, the Calvin cycle, the oxidative and reductive TCA cycles, biosynthetic pathways for all amino acids, pyrimidines and purines, the S-adenylsyl methionine (SAM) cycles I and II, the methyl alkane reductase pathway (North et al., 2020), sulfate assimilation, and generic RNA, DNA and protein synthesis pathways. The model represents an engineered species that is predicted to not need the ethylmalonyl CoA pathway due to the inclusion of a oxaloacetate decarboxylase/malic enzyme that converts oxaloacetate to pyruvate and CO_2_ and the inactivation of phospho-glycerate mutase. Consequently, all 3-phophoglycerate produced by the Calvin Cycle is stays within the Calvin cycle.

Boundary conditions (fixed species concentrations) consisted of concentrations of nutrients (ethanol, diphosphate, orthophosphate, sulfate and ammonia), cell waste products (CO_2_, H_2_, ethylene), ATP derivatives to set up the energy gradient due to photoheterotrophic growth (ATP, ADP, AMP), internal redox pairs (NAD+, NADH, NADP+, NADPH, reduced and oxidized ferredoxin, reduced and oxidized thioredoxin), folates (N10-formyltetrahydrofolate, 5-methyltetrahydrofolate, 5,10-methylenetetrahydrofolate, 7,8-dihydrofolate and tetrahydrofolate), and other internal metabolites for which scavenging pathways were not modeled (coenzyme A, (4S)-4,5-dihydroxypentan-2,3-dione, adenine, adenosine, adenosine-3’,5’-bisphosphate).

The standard free energy of reaction for each reaction was calculated with eQuilibrator, version 0.3.1. The free energies for the methyl alkane reductase pathway, the SAM cycles I & II, the ferredoxin oxidoreductases for 2-oxoglutarate and pyruvate, DNA, RNA and protein polymerization reactions were not available from eQuilibrator and were estimated manually from half-reactions reactions or overall reactions for the respective pathways. If estimates for some individual reactions were available for these pathways, they were adjusted accordingly, otherwise the standard free energy change for the pathways was divided equally among the constituent reactions. Details are included in the computational notebook available as supplemental material.

The model was constructed from a custom *R. rubrum* BioCyc database at PNNL. The draft model is available as a Jupyter notebook along with the necessary auxillary files for running the notebook in King and Cannon (2023).

## 3 Results

In order to place the results in the proper context, a brief overview of the metabolism of *R. rubrum*, the subject of the biomass optimization, is necessary. *R. rubrum* is capable of growing both photoautotrophically, meaning that it only uses CO_2_ as a carbon source, or photoheterotrophically, meaning that it can carry out photosynthesis while simultaneously assimilating organic carbon. In this study, we are modeling photoheterotropic growth, in which energy is acquired through photosynthesis while carbon is mostly acquired by uptake and assimilation of ethanol.

During photosynthesis, *R. rubrum* uses the Calvin cycle to regenerate chemical precursors for the assimilation of CO_2_. The Calvin cycle includes reactions shared with the non-oxidative branch of the pentose phosphate cycle, some of which can act as an alternate route in the Calvin Cycle. The two routes in the Calvin cycle differ in that one route produces erythrose-4-phosphate and the other route consumes erythrose-4-phosphate. Erythrose-4-phosphate is a key metabolite for the synthesis of vitamin B6, pyridoxal-5’-phosphate, an essential cofactor in amino acid, and hence protein, synthesis. Therefore, it is important that during growth, erythrose-4-phosphate not be entirely used as a Calvin cycle precursor.

We define the biomass pathways to be optimized as the pathways regarding the incorporation of amino acids into proteins and the incorporation of nucleotide triphosphates (NTPs) into RNA and DNA. The synthesis of amino acids and NTPs from precursors, however, are not included in the biomass pathways. During growth, proteins, RNA and DNA can be simultaneously synthesized, although RNA and DNA synthesis compete for both available energy and molecular precursors, NTPs and deoxynucleotide triphosphates (dNTPs), respectively. *R. rubrum* is known to have a cell cycle in which DNA can only be synthesized during the S phase, while RNA is synthesized continuously throughout the cell cycle except during mitosis. Whether DNA is synthesized depends on the redox state of the cell, specifically, the ratio of NADP to NADPH.

### 3.1 Controlling concentrations

Because some metabolites have highly favorable free energies of formation or standard chemical potentials, their concentrations can rise to dangerously high levels if not controlled (Atkinson, 1977). The primary role of control in a biological system must be to maintain viability. Metabolism will not operate if the concentrations of metabolites becomes so great that the cytoplasm becomes too viscous for diffusion-reaction processes to occur. We previously showed that concentrations of metabolites can be controlled by reducing key reaction fluxes through the activities of the respective enzymes identified by concentration control coefficients from Metabolic Control Analysis. In this approach, described briefly in the Methods section and in detail in reference (Britton et al., 2020), sensitivity analyses are used to characterize the influence of an enzyme’s activity on the concentration of any of the models metabolites. If a metabolite concentration is too high, the enzyme with the most influence over that metabolite is chosen to be regulated. This process occurs until all metabolite concentrations are at physiological levels. The approach can be implemented either as an optimal control problem or using reinforcement learning. Analysis of central metabolism showed that both analyses produced similar results and identified known regulators of central metabolism.

In Table 1, we compare the regulation of enzymes that were identified as necessary to be controlled to maintain physiological levels of metabolites in the MCA predictions with those from the pathway-controlled optimization (PCO) predictions. Of course, in the PCO method, reactions are controlled both to maintain physiological levels and to maximize flux to the growth reactions, so any amount of regulation predicted by the PCO method may result from a combination of the two objectives of maximizing growth while constraining metabolite concentrations to physiological levels. Nevertheless, as shown in Table 1, the PCO predicted regulation of reactions is consistent with the MCA predicted regulation of reactions.

**TABLE 1:** Reactions regulated in the MCA approach in which the goal was to control concentrations. Ten out of twelve of these reactions are also regulated in the pathway-controlled optimization approach.

Reaction	*J*_MCA_	*J*_PCO_	*α*_MCA_	*α*_PCO_
phosphoenolpyruvate + ADP = pyruvate + ATP	−5.85e+02	−9.60e+02	1.25e-01	1.00e+00
NAD+ + succinate = NADH + fumarate	−1.91e+03	−1.76e-08	1.17e-13	5.66e-28
acetaldehyde + NAD+ + H2O = acetate + NADH	4.32e+02	3.84e+03	1.16e-10	1.32e-11
acetate + ATP + CoA = acetyl-CoA + AMP + diphosphate	1.19e+03	4.69e+03	1.25e-01	1.00e+00
O-acetyl-L-homoserine + H2S = L-homocysteine + acetate	6.01e+01	4.15e-08	4.77e-07	3.26e-25
NH3 + L-glutamate + ATP = L-glutamine + ADP + orthophosphate	2.37e+02	6.23e-09	2.74e-10	3.98e-43
COA +2.0 AN_oxidized_ferredoxin + pyruvate = acetyl-CoA + CO2 + 2.0 A_reduced_ferredoxin	−2.83e+03	−5.07e+03	1.16e-10	1.24e-01
L-alanine + NAD+ + H2O = NH3 + pyruvate + NADH	−9.76e+01	−1.60e+02	4.88e-04	4.29e-10
ATP + L-aspartate + NH3 = AMP + L-asparagine + diphosphate	6.30e+02	1.60e+02	2.44e-04	2.09e-07
sulfate + ATP = adenosine-5’-phosphosulfate + diphosphate	1.95e+02	3.20e+02	2.47e-32	2.61e-05
ADP + A_reduced_thioredoxin = dADP + AN_oxidized_thioredoxin + H2O	3.49e+02	2.34e-08	1.28e-04	2.55e-16
IMP + NAD+ + H2O = NADH + XMP	6.30e+02	2.03e+03	7.35e-40	2.87e-06

The PCO method regulated ten out of twelve of the same reactions as the MCA method, suggesting that the PCO method reduced the activity of these reactions in order to maintain physiological levels of the metabolites. However, the observation that the flux in some of these reactions is now effectively zero may indicate that this subset of reactions were additionally shutdown to redirect flux to the growth reactions.

In addition, the PCO method may need to control more reactions than the MCA method, since reaction fluxes are also redistributed due to optimizing the growth reaction rates (discussed below). Decreased or increased reaction flux may result in higher reactant or product levels, respectively, such that the increased levels need to be controlled to keep them at physiological levels. The PCO approach regulated reactions that are not regulated in MCA and yet have non-zero fluxes. These are shown in Table 2. Presumably, these additional reactions need to be regulated to maintain metabolite concentrations at physiological levels in the new regime.

**TABLE 2:** Reactions regulated in the PCO method but not in the MCA method and that have significant reaction flux in the PCO method. Presumably these reactions are regulated to control metabolite concentrations.

Reaction	*J*_MCA_	*J*_PCO_	*α*_MCA_	*α*_PCO_
L-glutamine + 2-oxoglutarate + NADPH = 2 L-glutamate + NADP+	3.38e+02	−1.28e+03	1.00e+00	3.18e-17
3-phospho-D-glycerate + NAD+ + 3-phosphooxypyruvate + NADH	−3.41e+00	−4.27e+02	1.00e+00	3.61e-02
3-phospho-L-serine + 2-oxoglutarate = L-glutamate + 3-phosphooxypyruvate	3.41e+00	4.27e+02	1.00e+00	2.78e-02
3-phospho-L-serine + H2O = L-serine + orthophosphate	−3.41e+00	−4.27e+02	1.00e+00	1.27e-02
L-serine + L-homocysteine = L-cystathionine + H2O	−3.75e+01	−1.60e+02	1.00e+00	3.07e-02
O-succinyl-L-homoserine + L-cysteine = L-cystathionine + succinate	3.75e+01	1.60e+02	1.00e+00	1.80e-02
L-homoserine + succinyl-CoA = O-succinyl-L-homoserine + CoA	3.75e+01	1.60e+02	1.00e+00	1.56e-02
L-serine + Acetyl-CoA = O-Acetyl-L-serine + CoA	1.35e+02	3.20e+02	1.00e+00	9.25e-02
(2R)-2,3-dihydroxy-3-methylbutanoate = 3-methyl-2-oxobutanoate + H2O	1.95e+02	3.20e+02	1.00e+00	3.93e-02
(2R)-2,3-dihydroxy-3-methylbutanoate + NADP+ = (S)-2-acetolactate + NADPH	−1.95e+02	−3.20e+02	1.00e+00	1.38e-03
2 pyruvate = (S)-2-acetolactate + CO2	1.95e+02	3.20e+02	1.00e+00	1.30e-08
sulfate + ATP adenosine-5’-phosphosulfate + diphosphate	1.95e+02	3.20e+02	1.00e+00	5.82e-08
adenosine-5’-phosphosulfate + ATP = 3’-phosphoadenylyl-sulfate + ADP	1.95e+02	3.20e+02	1.00e+00	2.77e-06
H2S + 3 NADP+ + 3 H2O = sulfite +3 NADPH	−1.95e+02	−3.20e+02	1.00e+00	8.18e-05
oxaloacetate = CO2 + pyruvate	−1.66e+03	−3.15e+03	1.00e+00	1.65e-04
ATP + GMP = ADP + GDP	6.30e+02	2.03e+03	1.00e+00	8.39e-04
ATP + L-glutamine + H2O+ XMP = AMP + L-glutamate + GMP + diphosphate	6.30e+02	2.03e+03	1.00e+00	1.12e-05

### 3.2 Increasing growth

**FIGURE 1:**
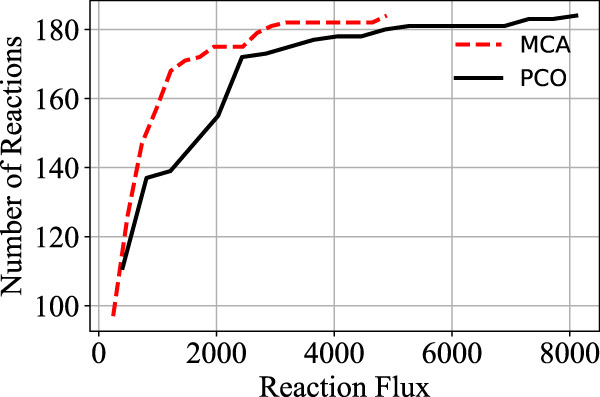
Comparison of the cumulative distribution of the reaction fluxes for the MCA model and the PCO model. Flux through the growth reactions are maximized by turning reaction fluxes down but not off.

**FIGURE 2:**
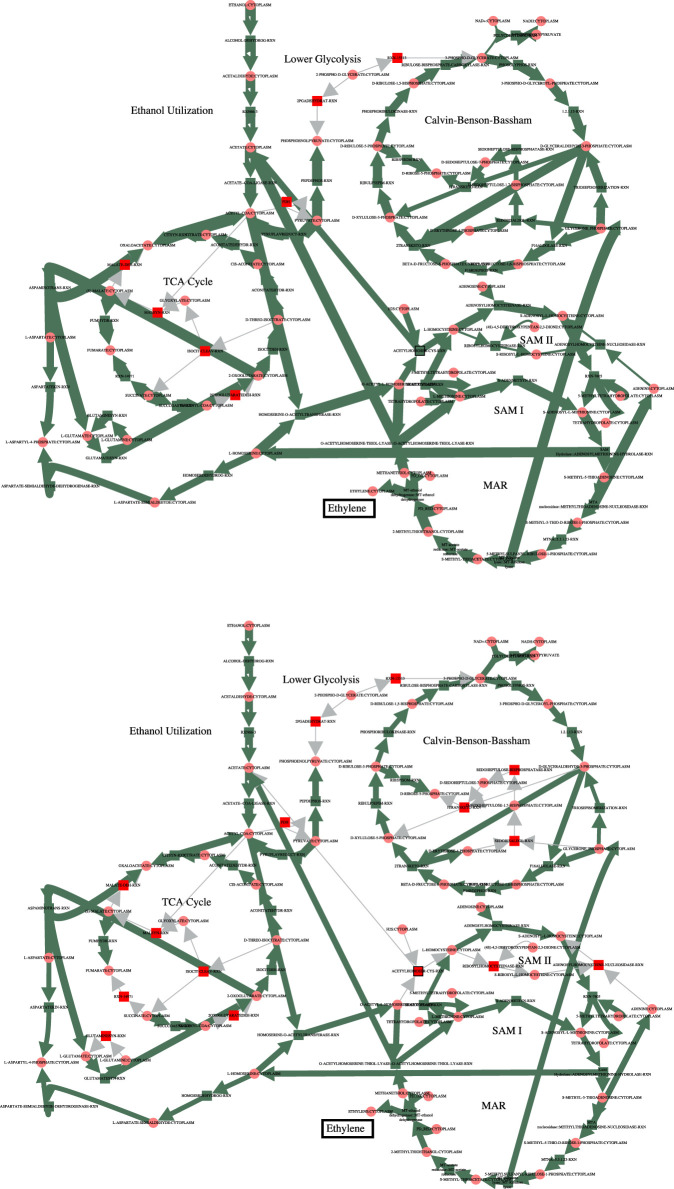
Comparison of the fluxes through central metabolism for the MCA model (top) and the PCO model (bottom). Flux through the growth reactions are maximized by turning reaction fluxes down but not off. The decreased flux through central metabolism in the PCO model reflects the same decrease shown below the red line for the MCA model in Figure 1.

In order to increase flux through the growth reactions in accordance with the PCO objective function, additional reactions are regulated to redirect flux to that end. The cumulative value of all the reaction fluxes for both the MCA-regulated reactions and the PCO-regulated reactions are shown in Figure 1. Some reactions in the PCO model are indeed reduced and some are increased, with the overall effect being a large increase in absolute rates of a subset of reactions. In general, more flux is directed to biosynthetic reactions, with the net result being a redirection of flux away from central metabolism in comparing the MCA-derived flux distribution to the PCO-derived flux distribution, as indicated in the flux maps for the two models in Figure 2.

The reactions with the highest fluxes from the PCO approach are shown in Table 3. The reaction with the highest absolute flux in the PCO regulated set of reactions is the RNA synthesis reaction, which increased to 8.11 × 10^3^, a more than 7-fold increase as compared to the MCA regulated model for controlling only metabolite levels. Likewise, the reaction for protein synthesis is included in this group. Both of these reactions are part of the growth reaction set used in the objective function. The other ten reactions with the highest flux are all involved in uptake and processing of the carbon source, ethanol. Much of the carbon from ethanol flows into fumarate and then into the pathways for synthesis of the pyrimidine nucleosides, uridine triphosphate (UTP) and cytidine triphosphate (CTP), precursors of RNA synthesis. In contrast the purine nucleosides are readily available from the already high levels of ATP.

**TABLE 3:** Top 12 reactions from the constrained optimization that have the highest fluxes. The reaction that has the highest flux in the constrained optimization is the growth reaction for RNA synthesis. Likewise, the reaction for protein synthesis is included in this group. Other reactions with the highest flux are all involved in uptake and processing of the carbon source, ethanol.

Reaction	*J*_MCA_	*J*_PCO_	Flux ratio
oxaloacetate + L-glutamate = L-aspartate + 2-oxoglutarate	−7.31e+02	−2.61e+03	3.57e+00
oxaloacetate = CO2 + pyruvate	−1.66e+03	−3.15e+03	1.89e+00
Amino Acids = protein	1.95e+03	3.20e+03	1.64e+00
GTP + IMP + L-aspartate = adenylo-succinate + GDP + orthophosphate	−1.32e+03	−3.57e+03	2.72e+00
adenylo-succinate = AMP + fumarate	−1.32e+03	−3.57e+03	2.72e+00
acetaldehyde + NAD+ + H2O = acetate + NADH	4.32e+02	3.84e+03	8.88e+00
ethanol + NAD+ = acetaldehyde + NADH	8.23e+02	4.48e+03	5.45e+00
acetate + ATP + CoA = acetyl-CoA + AMP + diphosphate	1.19e+03	4.69e+03	3.94e+00
CoA +2.0 AN_oxidized_ferredoxin + pyruvate = acetyl-CoA + CO2 + 2.0 A_reduced_ferredoxin	−2.83e+03	−5.07e+03	1.79e+00
(S)-malate = fumarate + H2O	4.89e+03	7.15e+03	1.46e+00
(S)-malate + NADP+ = NADPH + oxaloacetate	−4.89e+03	−7.15e+03	1.46e+00
NTPs = RNA	1.13e+03	8.11e+03	7.20e+00

Reactions that were effectively shutdown are shown in Table 4. As can be seen at the bottom of the table, DNA synthesis was minimized by significantly down regulating the synthesis of dNTPs, making more NTPs available for RNA synthesis. In addition, the alternate pathway in the Calvin cycle that consumes erythrose-4-phosphate is shutdown (top three reactions in Table 4), making more erythrose-4-phosphate available for synthesis of vitamin B6, a necessary component for production of the branched chain amino acids tryptophan and tyrosine.

**TABLE 4:** Reactions regulated in the PCO method but not in the MCA method and that have insignificant reaction flux in the PCO method. Presumably these reactions are shut down to redirect mass flow to the growth reactions.

Reaction	*J*_MCA_	*J*_PCO_	*α*_MCA_	*α*_PCO_
D-sedoheptulose-7-phosphate + D-glyceraldehyde-3-phosphate = D-ribose-5-phosphate + D-xylulose-5-phosphate	1.84e+01	−2.90e-09	1.00e+00	1.19e-10
D-sedoheptulose-1,7-bisphosphate + H2O = D-sedoheptulose-7-phosphate + orthophosphate	1.84e+01	−2.90e-09	1.00e+00	1.24e-10
glycerone_phosphate + D-erythrose-4-phosphate = D-sedoheptulose-1,7-bisphosphate	1.84e+01	−2.90e-09	1.00e+00	1.30e-10
2-phospho-D-glycerate = phosphoenolpyruvate + H2O	−1.78e-15	2.16e-13	1.00e+00	1.30e-15
acetyl-CoA + glyoxylate + H2O = COA + (S)-malate	3.55e-15	1.56e-12	1.00e+00	6.97e-07
S-adenosyl-L-homocysteine + H2O = S-ribosyl-L-homocysteine + adenine	3.68e+00	2.09e-07	1.00e+00	5.93e-08
S-ribosyl-L-homocysteine = L-homocysteine + (4S)-4,5-dihydroxypentan-2,3-dione	3.68e+00	2.09e-07	1.00e+00	6.05e-08
L-glutamine + L-aspartate + ATP + H2O = L-glutamate + L-asparagine + AMP + diphosphate	−5.32e+02	−6.16e-09	5.00e-01	9.45e-32
acetyl-CoA + L-glutamate = N-acetyl-L-glutamate + COA	3.55e-15	8.28e-12	1.00e+00	6.68e-44
ATP + dTMP = ADP + dTDP	3.49e+02	2.34e-08	1.00e+00	1.62e-10
ATP + L-glutamine + UTP + H2O = ADP + CTP + L-glutamate + orthophosphate	−2.56e-01	−5.10e-09	1.00e+00	4.12e-29
CTP + H2O = CDP + orthophosphate	3.32e-02	2.34e-08	1.00e+00	8.97e-10
CDP + A_reduced_thioredoxin = dCDP + AN_oxidized_thioredoxin + H2O	3.32e-02	2.34e-08	1.00e+00	9.26e-10
ATP + dCDP = ADP + dCTP	3.32e-02	2.34e-08	1.00e+00	9.68e-10
ATP + dTDP = ADP + dTTP	3.49e+02	2.34e-08	1.00e+00	2.06e-10
UDP + A_reduced_thioredoxin = dUDP + AN_oxidized_thioredoxin + H2O	6.97e+02	2.34 e-08	1.00e+00	8.51e-15
ATP + dUDP = ADP + dUTP	6.97e+02	2.34 e-08	1.00e+00	2.35e-14
dUTP + H2O = dUMP + diphosphate	3.49e+02	2.34e-08	1.00e+00	1.13e-10
ATP + dADP = ADP + dATP	3.49e+02	2.34e-08	1.00e+00	1.13e-11
GDP + A_reduced_thioredoxin = dGDP + AN_oxidized_thioredoxin + H2O	3.49e+02	2.34e-08	1.00e+00	4.33e-12
ATP + dGDP = ADP + dGTP	3.49e+02	2.34e-08	1.00e+00	4.80e-11
dNTPs = DNA	6.97e+02	4.68e-08	1.00e+00	4.88e-09

## 4 Discussion

### 4.1 Biological discussion

The PCO method we present here agrees with the majority of the regulation imposed by the MCA method for maintaining solvent capacity of the cell, which was shown previously to agree with known points of regulation from experiments as reported in the literature Britton et al. (2020). However, the consideration of growth as an additional objective for regulation in our PCO approach produced significant additional regulation in line with adjusting flux through growth pathways. The inclusion of the consideration of growth is likely to be crucial for a complete understanding of regulation in cells.

While this study uses a limited model (184 reactions and 204 metabolites), that DNA synthesis was turned off, as it is during most of the cell cycle, is interesting, and presumably was done so in order to maximize RNA synthesis. Further evaluation with a more complete model is needed. It is not clear if under low NADP/NADPH conditions that DNA synthesis is likewise maximized by reducing RNA synthesis.

Of course, it will be critical to test the results of the model against experimental observations in order to draw firm biological conclusions. In this regard, previous studies of the metabolic dynamics of *R. rubrum* using Metabolic Flux Analysis (McCully et al., 2020), an experimental method to derive fluxes from observations of isotope distributions, will provide a good test of the regulation model.

If successful, the incorporation of thermodynamics into the optimization of growth with the PCO approach developed here, along with the ability to infer kinetic parameters from maximum path entropy distributions, will provide a way to further explore the relationship between natural selection and thermodynamics in detail. Ideally, these capabilities will allow the study of the expression patterns and activities of enzymes at various stages in the cell cycle.

### 4.2 Optimization discussion

One of the biggest challenges in solving the PCO problem is the range of the variable values. For the solutions presented here both metabolites concentrations and activity coefficients for reactions vary over many orders of magnitude. To make the problem more numerically tractable our formulation in essence seeks to do computations with respect to the log of the variables to mitigate the large discrepancies in scale. This is relatively straightforward with respect to the metabolite concentrations in part because they must be positive. However, to handle the flux we must separate out its magnitude and sign which complicates the formulation. It is important to note that our approach does not strictly adhere to the requirements of the IPOPT solver, namely, that the objective and constraints be twice continuously differentiable. In particular, both constraint (27a) and (30h) violate this condition.

For the constraint (27a), the original expression (19c) it is derived from is twice continuously differentiable but resulted in numerical errors. By introducing the form (20) and using the approximation (28) to the signum function a cusp is introduced into the first derivative at zero and error is introduced in an interval about the origin determined by the parameters *λ* and *ϵ*. We choose *λ* and *ϵ* to shrink this interval such that the true derivative is closely approximated at all points evaluated by the solver. We found this choice to produce more accurate solutions and better convergence behavior than using twice continuously differentiable approximations of the signum function or other forms for (19c). It may be possible to further improve performance by supplying the solver directly with alternative derivative computations rather than relying on automatic differentiation, particularly if solutions approach the origin.

The relaxed formulation (30) also uses the approximation (28) to the signum function in the constraint (30h) specifying the value of the ‘switching’ variable *u*. Here choices of *λ* and *ϵ* are made such that the right hand side of the constraint is effectively constant and partials with respect to the flux will evaluate to zero, in order to hold *u* constant at 1 or −1 so long as the sign of the flux remains the same. This formulation captures the problem well when the sign on the fluxes are in the optimal direction but it is unclear to what extent it constrains the domain reachable by the solver from an arbitrary initial condition. We also evaluated solutions including an explicit computation of the activity coefficients as in (20) but found that convergence of the solver required setting a lower bound on regulation of roughly 1 × 10^−9^, and solutions had a significantly lower objective value than were found using the implicit regulation formulation (30).

Due to the potential restrictions on the solutions that can be obtained with our approach using the relaxed formulation (30) we will consider methods to more thoroughly explore the feasible set in future work including stochastic search methods. Of course a more complete exploration of the space of solutions can come at significant computational cost and if other methods perform similarly to the approach we present here, then it may have a clear practical advantage in that the times-to-solution will likely be much faster. Additionally, as discussed in Section 2.4.3 solutions are not necessarily unique. Methods to explore and characterize the set of equivalent solutions, that is solutions that can achieve the same objective value, will allow for a greater understanding of the flexibility that may be possible in controlling reaction fluxes and metabolite concentrations while achieving high growth rates.

## Data Availability

The original contributions presented in the study are included in the article/Supplementary Material, further inquiries can be directed to the corresponding author.

## Supplementary material

Supplementary Data Sheet S1 Equilibrium constants.

Supplementary Data Sheet S2 Metabolite concentrations.

Supplementary Data Sheet S3 Stoichiometric Matrix.
